# Role of Interfacial Processes in Accelerated Reactions in Nano- and Microdroplets

**DOI:** 10.1021/acs.jpca.5c03287

**Published:** 2025-07-04

**Authors:** Shu Yang, Meng Li, Justin Wang, Vicki H. Grassian, Satish Kumar, Cari S. Dutcher

**Affiliations:** † Department of Mechanical Engineering, 5635 University of Minnesota, Minneapolis, Minnesota 55455, United States; ‡ Department of Chemistry and Biochemistry, 8784 University of California San Diego, La Jolla, California 92093, United States; § Department of Chemical Engineering and Materials Science, University of Minnesota, Minneapolis, Minnesota 55455, United States

## Abstract

Reaction kinetics can be significantly accelerated in microconfinement, where interfacial processes play a critical role. We developed a kinetic model describing diffusion, adsorption, evaporation, partitioning, and surface reactions in a microdroplet. Tensiometry measurements are utilized to parametrize the adsorption kinetics using a Langmuir adsorption model. The model quantitatively reproduces previous experimental measurements of the concentration and droplet size evolution during the condensation reaction of pyruvic acid (PA) to zymonic acid (ZA) in microdroplets. We further generalize the model to systems where the interplay between reaction and transport processes varies with droplet size from nanometer to millimeter scales, leading to diverse kinetic behaviors unique to the droplet environment. Notably, we observe an intriguing competition between evaporation and reaction that determines the optimal droplet size. While smaller droplets exhibit faster reaction rates due to the dominance of surface reactions, they also experience higher PA evaporation rates, leading to more PA being consumed via evaporation rather than the reaction. These findings offer insights into the complexity of microdroplet reaction kinetics and elucidate general mechanisms for understanding processes that control the reaction kinetics in droplets over a wide range of length scales.

## Graphical abstract

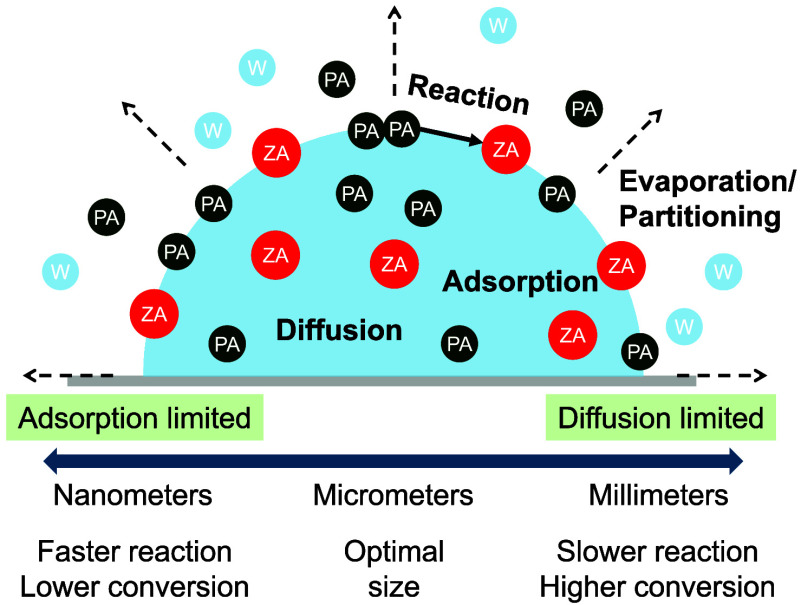

## 1 Introduction

Microdroplets facilitate unique environments that significantly accelerate chemical reactions compared to bulk-phase environments.^1–15^ The accelerated reactions in aqueous droplets, ranging from the nanometer to millimeter scales, play a critical role in aerosol reactions in atmospheric chemistry,^9,16–19^ as well as in microreactors in biochemical screening^20,21^ and emulsion polymerization in polymer synthesis.^22,23^ One notable example is the reaction of pyruvic acid (PA), a small α-keto acid abundant in aerosols and crucial in secondary organic aerosol formation via photochemical processes.^24–26^ In microdroplets, PA was found to undergo condensation reactions to form zymonic acid (ZA), a reaction that does not occur readily in the bulk phase, suggesting that the reaction occurs only at the air–water interface.^6,18^

We recently developed a diffusion-reaction-partitioning model to elucidate the reaction kinetics of PA to ZA in microdroplets.^18^ The model quantitatively predicts the evolution of droplet size and composition over time under various environmental conditions and estimates the reaction rate coefficients by fitting experimental data. The size-dependent PA reaction kinetics were observed to exhibit a sigmoidal pattern,^6^ a feature often indicative of possible autocatalysis, where the product catalyzes its own formation.^27,28^ While the model best fits the data when incorporating an autocatalytic step, our results suggest that the gas-phase partitioning (including PA evaporation and water partitioning) alone can also produce sigmoidal kinetics, even in the absence of autocatalysis.^18^ Overall microdroplet reactivity is governed by both surface reactions and gas-phase partitioning, highlighting the critical role of interfacial physical processes in reaction kinetics.^18^ The work also clearly demonstrates that microdroplet kinetic analyses differ in significant ways from established methods for bulk solutions.^18^

However, our previous model did not take into account the adsorption and desorption of PA and ZA between the interface and the bulk phase.^18^ Instead, it treats surface concentrations as equivalent to bulk concentrations, which may lead to inaccuracies. Many organic species strongly adsorb to interfaces,^29^ and increased interfacial concentrations have been identified as a key factor in accelerated reactivity in microdroplets.^29–32^ Although there were previous kinetic models considering adsorption processes in microdroplet reactions,^18,33,34^ the impact of gas-phase partitioning was overlooked, which can significantly influence microdroplet reactivity. The complexity of surface reactions—shaped by diffusion, adsorption, evaporation, and gas-phase partitioning, necessitates a more sophisticated model for accurate characterization. Understanding these processes, each of which exhibits a distinct characteristic length scale, is crucial for capturing diverse kinetic behaviors across different compartment sizes and their impact on reaction kinetics in various applications.^35,36^

This work develops a theoretical kinetics-based model to investigate accelerated reactivity in microdroplets. Using the PA condensation reaction as an example, we examine the complex interplay among diffusion, adsorption, evaporation, and surface reactions in droplets ranging from nanometer to millimeter scales. Our framework reveals fundamental principles governing microdroplet reactivity and shows that the relative importance of interfacial processes (i) varies significantly with droplet size and (ii) can be predicted by comparing the characteristic timescales of these processes. These findings offer insights into efficient chemical processing and natural phenomena, guiding the selection and control of reactions in droplets over a wide range of length scales.

## 2 Methods

This section presents a mathematical model that characterizes the dynamics at the surface of a hemispherical droplet containing reactant PA and product ZA, as shown schematically in Figure 1. This model describes the diffusion of PA and ZA in accordance with Fick’s law, while adsorption is described by the Langmuir model. Additionally, the model accounts for PA evaporation and water partitioning, with ZA considered nonvolatile. PA and ZA participate in autocatalytic reactions at the air–water interface^6,18^

1 $$2PA\overset{k_{f1}}{\underset{k_{b1}}{⇌}}\mathrm{ZA}$$

2 $$2\mathrm{PA}\overset{k_{f2},\mathrm{ZA}}{\underset{k_{b2}}{⇌}}\mathrm{ZA}$$

Here *k*_f*i*_ and *k*_b*i*_ are the forward and backward rate constants for the first (*i* = 1) and second (*i* = 2) reactions, respectively. The relative importance of diffusion, adsorption, evaporation, and reaction in determining the droplet composition in sizes ranging from nanometers to millimeters is discussed in Section 3.

**Figure 1:**
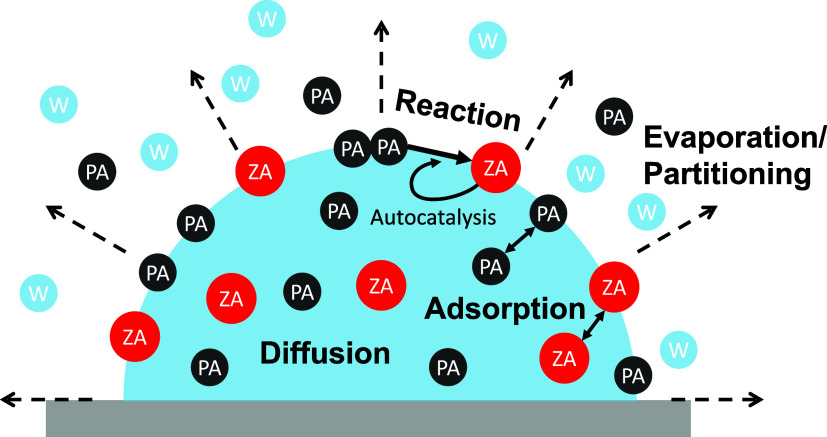
Relevant microdroplet processes in the kinetic model include the evaporation of PA and partitioning of water (W); adsorption of PA and ZA between the interface and the bulk; diffusion of PA and ZA; interfacial reactions of PA to form ZA. Schematic adapted with permission from our previous publication (ref 18), licensed under a Creative Commons Attribution 3.0 Unported License, with permission from the Royal Society of Chemistry.

The model presented here builds upon our previous diffusion-reaction-partitioning framework,^18^ which was developed based on experimental setups designed by Li et al.^6^ In these experiments, a microdroplet containing PA was placed in an environmental cell maintained at a constant temperature and a stable relative humidity (RH), where it retained a nearly hemispherical shape with a radius *R* on the hydrophobic substrate.^6^ Assuming symmetry in the polar and azimuthal directions, we restrict our problem to radial coordinate *r* and time *t*. Here we incorporate the adsorption process of PA and ZA and calculate their bulk concentrations *C*_*X*_(*r*, *t*) and interfacial concentrations Γ_*X*_(*r*, *t*), where the subscript *X* refers to either PA or ZA. Note that in the following derivations, molar concentrations *C*_*X*_ and Γ_*X*_ (in moles per cubic meter of solution) are employed. The interfacial concentration is converted from units of mol m^–2^ to bulk concentration units (mol m^–3^) by adopting a surface thickness δ = 1 nm, as suggested by molecular dynamics simulations.^34,37^ The results are later converted to molarities *m*_*X*_ (bulk) and γ_*X*_ (interface) (in moles per kilogram of water) to allow a direct comparison with the experimental data. The key nomenclature is summarized in Table 1.

**Table 1:** Key Nomenclature^a^

parameter	nomenclature	units
*C*_*X*_	bulk concentration	mol m^–3^
Γ_*X*_	interfacial concentration	mol m^–3^
*V*_*X*_	volume	m^3^
σ_*X*_	surface tension	N m^–1^
*m*_*X*_	bulk concentration	mol kg^–1^
γ_*X*_	interfacial concentration	mol kg^–1^

a The subscript *X* can be PA or ZA for all six parameters. For Volume (*V*_*X*_), the subscript *X* can be PA, ZA, or W (water).

In the droplet (0 < *r* < *R*(*t*)), the bulk concentrations follow Fick’s diffusion equation

3 $$\frac{\partial C_{X}}{\partial t}=\frac{D_{X,l}}{r^{2}}\;\frac{\partial }{\partial r}\left(r^{2}\frac{\partial C_{X}}{\partial r}\right)$$

where *D*_*X*,l_ is the diffusion coefficient of substance *X* in the liquid phase. The parameters used in the governing equations are given in Table 2. Mass conservation for a total number of molecules of substance *X* reads

4 $$\frac{d}{dt}\int_{0}^{R\left(t\right)}C_{X}d\left(\frac{2\pi }{3}r^{3}\right)+\frac{d}{dt}\left(\Gamma_{X}2\pi R{\left(t\right)}^{2}\delta \right)=J_{X,\mathrm{rxn}}2\pi R{\left(t\right)}^{2}\delta +\frac{dV_{X,\mathrm{evp}}}{dt}\;\frac{1}{\nu_{X}}$$

The two terms on the left-hand side represent the change in the total number of molecules of substance *X* in the bulk and at the surface, respectively. This change corresponds to the sum of the contributions from the surface reaction (first term on the right-hand side) and evaporation (second term on the right-hand side). Here d*V*_*X*,evp_/d*t* is the rate of volume change of *X* due to evaporation and ν_*X*_ is the molar volume of *X*, where d*V*_ZA,evp_/d*t* = 0 (involatile) and d*V*_PA,evp_/d*t* will be given in ([Disp-formula eq12]). The rate of change of Γ_*X*_ due to the reaction *J*_*X*,rxn_ can be calculated from ([Disp-formula eq1]) and ([Disp-formula eq2])­

5 $$J_{\mathrm{PA},\mathrm{rxn}}=-2\left(k_{f1}^{\prime }\Gamma_{\mathrm{PA}}^{2}+k_{f2}^{\prime }\Gamma_{\mathrm{PA}}^{2}\Gamma_{\mathrm{ZA}}-k_{b1}^{\prime }\Gamma_{\mathrm{ZA}}-k_{b2}^{\prime }\Gamma_{\mathrm{ZA}}^{2}\right)$$

6 $$J_{\mathrm{ZA},\mathrm{rxn}}=\left(k_{f1}^{\prime }\Gamma_{\mathrm{PA}}^{2}+k_{f2}^{\prime }\Gamma_{\mathrm{PA}}^{2}\Gamma_{\mathrm{ZA}}-k_{b1}^{\prime }\Gamma_{\mathrm{ZA}}-k_{b2}^{\prime }\Gamma_{\mathrm{ZA}}^{2}\right)$$

where *k*_f*i*_^′^ and *k*_b*i*_^′^ are reaction rate coefficients associated with molar concentration.

**Table 2:** Values of Quantities Used in the Model at 295 K and 95% RH^a^

parameter	value
*D*_PA,l_	2.5 × 10^–10^ m^2^ s^–1^
*D*_ZA,l_	1.8 × 10^–10^ m^2^ s^–1^
Γ_PA,∞_	3.887 × 10^–6^ mol m^–2^
Γ_ZA,∞_	2.016 × 10^–6^ mol m^–2^
κ_PA_	8.153 × 10^–3^ m^3^ mol^–1^
κ_ZA_	3.847 × 10^–3^ m^3^ mol^–1^
*k*_d,PA_	90 s^–1^
*k*_d,ZA_	90 s^–1^
*P*_sat,PA_	107 Pa
*D*_PA,g_	9.8 × 10^–6^ m^2^ s^–1^
ν_PA_	7.0 × 10^–5^ m^3^ mol^–1^
ν_ZA_	1.3 × 10^–4^ m^3^ mol^–1^
ν_w_	1.8 × 10^–5^ m^3^ mol^–1^

a The estimations for *D*_*X*,l_, *D*_PA,g_, and *P*_sat,PA_ are adapted from our previous publication (ref 18), licensed under a Creative Commons Attribution 3.0 Unported License, with permission from the Royal Society of Chemistry.

At *r* = 0, there is a no-flux boundary condition

7 $$\frac{\partial C_{X}}{\partial r}=0$$

Using Leibniz’s rule and substituting ([Disp-formula eq4]) into ([Disp-formula eq3]) yields boundary conditions at the interface *r* = *R*(*t*)­

8 $$D_{X,l}\frac{\partial C_{X}}{\partial r}=-C_{X}\frac{dR}{dt}+J_{X,\mathrm{ads}}$$

and evolution equations for interfacial concentrations

9 $$\frac{d\Gamma_{X}}{dt}=-\Gamma_{X}\frac{2}{R}\;\frac{dR}{dt}+J_{X,\mathrm{rxn}}-J_{X,\mathrm{ads}}+\frac{dV_{X,\mathrm{evp}}}{dt}\;\frac{1}{2\pi R^{2}\delta \nu_{X}}$$

The first terms on the right-hand sides of ([Disp-formula eq8]) and ([Disp-formula eq9]) represent the impact of droplet size variation on the bulk concentration and interfacial concentration, respectively. The adsorption flux *J*_*X*,ads_ is described by the Langmuir isotherm^38^

10 $$J_{X,\mathrm{ads}}=k_{a,X}C_{X}\left(1-\frac{\Gamma_{\mathrm{PA}}+\Gamma_{\mathrm{ZA}}}{\Gamma_{\infty }}\right)+k_{d,X}\Gamma_{X}$$

where *k*_a,*X*_ and *k*_d,*X*_ denote the adsorption and desorption rates of substance *X*. The maximum surface coverage of the PA and ZA mixture solution is denoted by Γ_∞_, which is approximated using a weighted average formula: Γ_∞_ = (Γ_∞,PA_ *C*_PA_ + Γ_∞,ZA_ *C*_ZA_)/(*C*_PA_ + *C*_ZA_). The ratio of adsorption rate to desorption rate κ_*X*_ = *k*_a,*X*_/*k*_d,*X*_ and the maximum surface coverage Γ_∞,*X*_ of PA and ZA are obtained (values listed in Table 2) by the fitting of surface tension σ_*X*_ (shown in Figure 2) using the Langmuir isotherm

11 $$\sigma_{X}=\sigma_{o}+R_{g}T\Gamma_{\infty ,X}\;\ln\left(1-\frac{\kappa_{X}c_{X}}{1+\kappa_{X}c_{X}}\right)$$

where σ_o_ is the surface tension of pure water at the air–water interface, *R*_g_ is the universal gas constant, and *T* = 295 K is temperature. The calculation of the adsorption flux ([Disp-formula eq10]) requires the values of both *k*_a,*X*_ and *k*_d,*X*_, while the surface tension fitting yields only their ratio κ_*X*_ = *k*_a,*X*_/*k*_d,*X*_. Note that desorption rate coefficients show minimal variation across different molecular structures and functional groups,^39^ prompting us to employ a median value of *k*_d,*X*_ = 90 s^–1^ for our calculations. In Section 3.1.2, the values of *k*_d,*X*_ and κ_*X*_ will be varied to assess their impact on the reaction kinetics.

**Figure 2:**
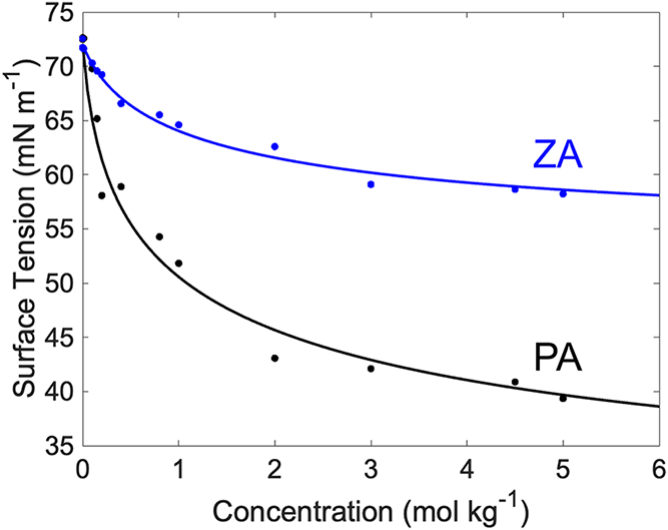
Fitting of the Langmuir equation to the surface tension data of PA and ZA as a function of the concentration at 295 K.

The surface tension measurements shown in Figure 2 were performed using an AquaPi Surface Tensiometer fitted with a PTFE sample cup and a DyneProbe. The tensiometer was calibrated with MilliQ water with resistivity >18.1 MΩ. Pyruvic acid (Sigma Aldrich, 98%) was obtained from Thermo Scientific (extra pure, nitrogen-flushed), and its purity was further confirmed by ^1^H NMR analysis prior to use. All tensiometry measurements were conducted on the same day and completed within 8 h following sample preparation. Zymonic acid was isolated following an experimental procedure outlined by Vaida and co-workers.^40^ All solutions were prepared in 20 mL scintillation vials with Teflon caps. Solutions of pyruvic acid were prepared by pipetting the acid directly into the vial and adding Milli-Q water to the desired concentration, while solutions of zymonic acid were prepared by weighing the acid in the vial, then adding Milli-Q water, and dissolving by agitation. Three solutions were independently prepared for each desired concentration of each acid species. Each solution was measured using the tensiometer three times, and the last two readings were averaged to provide a data point. Between the measurement of each solution, the tensiometer sample cup and probe were thoroughly rinsed with Milli-Q water, and the probe was heated directly in the flame of a butane torch for 20 s. The final data was obtained by averaging the data points across the three independent solutions of the same concentration.

The partial differential in equation ([Disp-formula eq3]) is subject to mass-conservation conditions ([Disp-formula eq8]) at the moving boundary *r* = *R*(*t*). In our previous work, we derived an expression for *R*(*t*), which is influenced by PA evaporation, PA and ZA reactions, and water partitioning.^18^ For gas-phase diffusion-controlled evaporation in a hemispherical droplet, the volume change resulting from the evaporation of PA is^41,42^

12 $$\frac{dV_{\mathrm{PA},\mathrm{evp}}}{dt}=-2\pi RD_{\mathrm{PA},g}\Delta \Gamma_{\mathrm{PA},g}\nu_{\mathrm{PA}}k_{\mathrm{evp}}$$

where *D*_PA,g_ represents the diffusivity of PA in the gas phase. The difference in vapor concentration between the droplet surface and the region far from it, based on the ideal gas law and Raoult’s law, is given by ΔΓ_PA,g_ = *x*_PA_ *P*_sat,PA_/*R*_g_ *T*. Here *P*_sat,PA_ is the PA saturation vapor pressure, and *x*_PA_ is the mole fraction of PA at the interface. Note that the PA partial pressure in the gas phase far from the droplet is zero as humidified N_2_ is passed through the environmental cell during the experiment, thereby removing the PA vapor. So ΔΓ_PA,g_ in ([Disp-formula eq12]) can be written as

13 $$\Delta \Gamma_{\mathrm{PA},g}=\frac{P_{\mathrm{sat},\mathrm{PA}}}{R_{g}T}\;\frac{\Gamma_{\mathrm{PA}}}{\Gamma_{\mathrm{PA}}+\Gamma_{\mathrm{ZA}}+\nu_{W}\left(1-\Gamma_{\mathrm{PA}}\nu_{\mathrm{PA}}-\Gamma_{\mathrm{ZA}}\nu_{\mathrm{ZA}}\right)}$$

In ([Disp-formula eq12]), the dimensionless correction factor *k*_evp_ = 0.6 accounts for the discrepancy between the model’s evaporation rates and experimental data, primarily due to nonideal mixing and the unknown activity coefficient of PA. We demonstrated that *k*_evp_ can be determined by fitting data from the induction period, during which evaporation dominates droplet size change and the reaction is minimal.^18^ Note that the temperature change in the droplet is neglected due to the temperature-controlled experimental setup. The droplet is placed on a temperature-controlled substrate within an environmental cell, both maintained at 295 K.

The volume change of species *X* caused by the reactions is

14 $$\frac{dV_{X,\mathrm{rxn}}}{dt}=2\pi R^{2}\delta \nu_{X}J_{X,\mathrm{rxn}}$$

Estimating water partitioning poses a challenge due to the unknown water activity at the interface. However, given that the water saturation pressure is ∼30 times higher than that of PA,^43,44^ it is reasonable to assume that water rapidly reaches equilibrium between the droplet and gas phase. As a result, at a constant RH, the partitioning of water is determined by Γ_PA_ and Γ_ZA_. According to the Zdanovskii-Stokes-Robinson mixing rule,^45^ a change of one mole of substance *X* results in a corresponding change of *k*_*X*_ moles of water. We have *k*_PA_ = 12.4 and *k*_ZA_ = 4.2 estimated from the molality of pure PA (4.5 mol kg^–1^, estimated from constant PA molality observed in the induction stage) and pure ZA (13.3 mol kg^–1^, estimated using the Aerosol Inorganic–Organic Mixtures Functional Groups Activity Coefficients model^46^) at 95% RH. Under this assumption, the water content change resulting from *X* content change is

15 $$\frac{dV_{W}}{dt}=\frac{dV_{X}}{dt}\;\frac{k_{X}\nu_{w}}{\nu_{X}}$$

Note that RH influences the model by modulating the *k*_*x*_ value in the model. If RH in the gas phase decreases, the water content in the droplet will also decrease, which leads to a reduction in *k*_*X*_. Using ([Disp-formula eq12])–([Disp-formula eq15]), the volume change of the droplet is

16 $$2\pi R^{2}\frac{dR}{dt}=\frac{dV_{\mathrm{PA},\mathrm{evp}}}{dt}\left(1+\frac{k_{\mathrm{PA}}\nu_{w}}{\nu_{\mathrm{PA}}}\right)+\frac{dV_{\mathrm{PA},\mathrm{rxn}}}{dt}\left(1+\frac{k_{\mathrm{PA}}\nu_{w}}{\nu_{\mathrm{PA}}}\right)+\frac{dV_{\mathrm{ZA},\mathrm{rxn}}}{dt}\left(1+\frac{k_{\mathrm{ZA}}\nu_{w}}{\nu_{\mathrm{ZA}}}\right)$$

The various processes occurring in the droplet are governed by coupled equations describing the evolution of interfacial concentrations ([Disp-formula eq9]), droplet size ([Disp-formula eq16]), and bulk concentrations ([Disp-formula eq3]) subject to boundary conditions ([Disp-formula eq7]) and ([Disp-formula eq8]). A coordinate transformation (*r*, *t*) → (η, τ) is applied to the governing equations to solve this moving boundary problem, where η = *r*/*R* and ∂τ/∂*t* = 1. The transformed spatial domain η ∈ (0, 1) is then discretized by using a centered second-order finite-difference method. Time integration is conducted with MATLAB’s ode15s solver. Convergence analysis shows that 100 grid points in the spatial domain provide sufficient accuracy.

We aim to use a minimal model to capture the key features of the reaction kinetics. However, several simplifying assumptions may introduce deviations from the experimental behavior. One key assumption is that of a hemispherical droplet geometry and a one-dimensional system, based on experimental observations showing that droplets maintain a near-hemispherical shape with contact angles consistently around 94 ± 4° throughout the process.^6^ While this approximation facilitates model tractability, it may be less accurate for smaller droplets or cases with strong substrate interactions, where deformation can alter surface area, curvature, and evaporation rates, potentially affecting kinetics. Another limitation involves the treatment of the activity coefficient of PA. To focus on the role of evaporation, we employ a fitted constant *k*_evp_ to approximate the influence of nonideal activity, rather than explicitly modeling its concentration dependence through thermodynamic expressions. The model assumes instantaneous equilibrium of the water content between the gas phase and the droplet. Potential delays in this equilibrium due to diffusion and adsorption limitations, nonideal interactions (e.g., hydrogen bonding), and other mass transfer constraints are neglected.

Finally, since our study spans droplet sizes from the nanometer to millimeter scale, additional physical effects may become important at the extremes. At larger scales, internal advection within the droplet could influence mass transport, but this is not accounted for in the current model. At the nanometer scale, both curvature and confinement effects may significantly impact the adsorption and evaporation behavior.

## 3 Results and Discussion

### 3.1 Kinetics of Chemical Reactions in Microdroplets

The kinetic model described in Section 2 quantitatively predicts the evolution of PA bulk concentration *m*_PA_ (at *r* = *R*/2) and droplet radius *R* at different initial radii *R*_o_, as shown in Figure 3. As previously reported, the size-dependent profiles of *m*_PA_ follow a sigmoidal pattern, where *m*_PA_ remains relatively constant during the induction period, followed by a sharp decline during the reaction phase, and then they eventually reach a plateau as the reaction and evaporation near completion.^6,18^ The initial PA concentration used in the experiments (*m*_PA,o_ = 4.5 mol kg^–1^; Figure 3) may approach or exceed the critical micelle concentration (CMC) (Figure 2), where micelle formation could reduce the effectiveness of the Langmuir adsorption model and lead to an overestimation of Γ_PA_. We retain the Langmuir model for simplicity at higher concentrations, but later calculations will use a lower *m*_PA,o_ well below the CMC. The reaction rate coefficients were optimized by minimizing the sum of squared residuals between the experimental and simulated concentration/radius–time data using MATLAB’s fminsearch function. The reaction rate coefficients estimated by our current model are of a similar order of magnitude to those estimated by the previous model, which does not include adsorption,^18^ suggesting that adsorption may not be a critical step in this particular system. The influence of adsorption on the reaction kinetics will be discussed later in Section 3.1.2.

**Figure 3:**
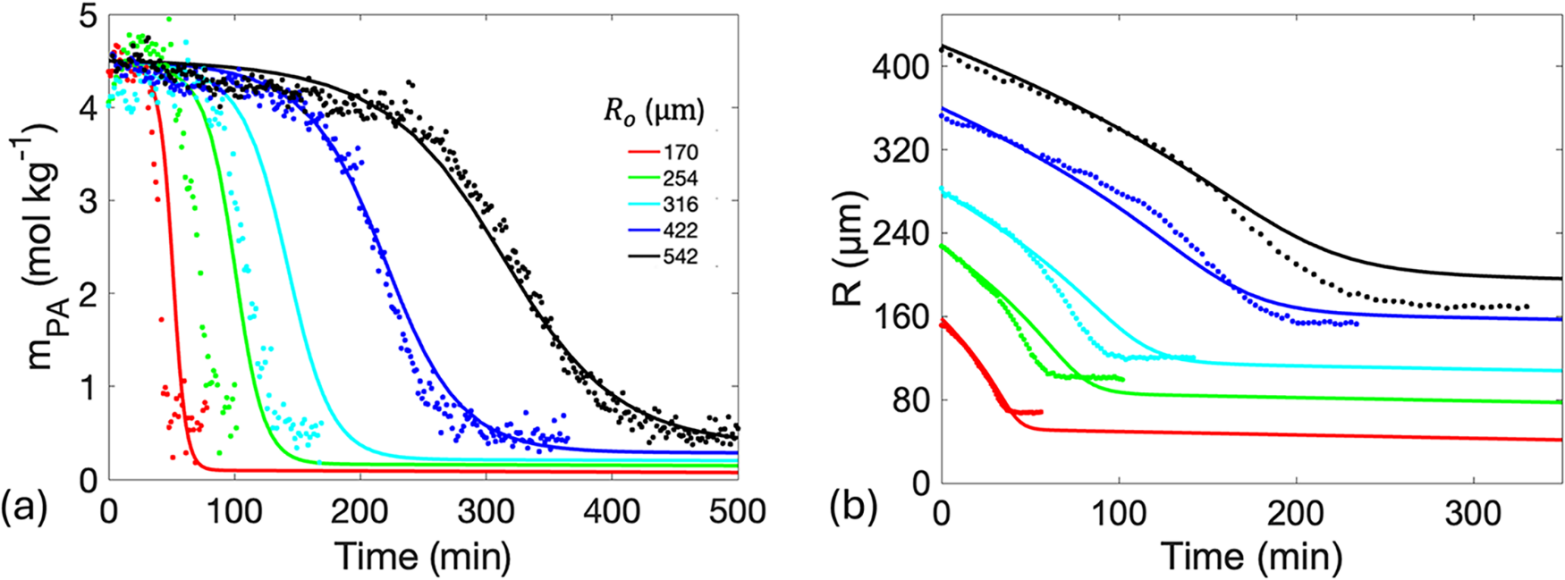
Model (solid lines) fits the experimental data points using reaction rate coefficients *k*_f1_ = 0.15 kg mol^–1^ s^–1^, *k*_f2_ = 2.9 kg^2^ mol^–2^ s^–1^, *k*_b1_ = 0.08 s^–1^, and *k*_b2_ = 0.04 kg mol^–1^ s^–1^. The time evolutions of (a) *m*_PA_ at *r* = *R*/2 and (b) *R* were examined at different *R*_o_. Curves increase in *R*_o_ from left to right. Experimental data reprinted with permission from our previous publication (ref 18), licensed under a Creative Commons Attribution 3.0 Unported License, with permission from the Royal Society of Chemistry.

The relative significance of diffusion, adsorption, evaporation, and reaction can be assessed by comparing their respective timescales, with the process that has the longest time scale serving as the rate-limiting step. Note that water partitioning is not discussed here because we reasonably assume that water reaches instantaneous equilibrium between the droplet and gas phase, as detailed in ([Disp-formula eq15]). The time scale for diffusion of PA from the bulk to the interface can be approximated using ([Disp-formula eq3])­

17 $$\tau_{\mathrm{dif}}=\frac{R_{o}^{2}}{D_{\mathrm{PA}}}$$

According to the Langmuir framework,^35,47^ the characteristic time scale for adsorption is

18 $$\tau_{\mathrm{ads}}=\frac{1}{k_{d}\left(\kappa c_{\mathrm{PA}}+1\right)}$$

The time scale for the evaporation of PA can be estimated using ([Disp-formula eq12])­

19 $$\tau_{\mathrm{evp}}=\frac{R_{o}^{2}}{E_{\mathrm{PA}}}x_{\mathrm{PA}}$$

where the evaporation coefficient *E*_PA_ = *D*_PA,g_ *P*_sat,PA_ (ν_PA_ + *k*_w_ν_w_)/*R*_g_ *T*, and *x*_PA_ is PA mole fraction. τ_evp_ equals the time required for the PA solution to completely evaporate in the absence of any reactions. The time scale for the surface reaction that changes PA bulk concentration can be approximated using ([Disp-formula eq7])­

20 $$\tau_{\mathrm{rxn}}=\frac{1+R_{o}/3\delta }{2k_{f1}\Gamma_{\mathrm{PA}}}$$

The relative importance of various transport processes—diffusion, adsorption, and evaporation—compared to chemical reactions can be evaluated using dimensionless quantities known as Damköhler numbers (Da). Damköhler numbers express the ratio of the characteristic reaction time scale to the timescales of the respective transport mechanisms, highlighting which transport processes may limit the reaction. The Damköhler numbers for each transport process are defined as follows

21 $$Da_{\mathrm{dif}}=\frac{\tau_{\mathrm{rxn}}}{\tau_{\mathrm{dif}}}=\frac{D_{\mathrm{PA}}\left(1+R_{o}/3\delta \right)}{2k_{f1}\Gamma_{\mathrm{PA}}R_{o}^{2}}$$

22 $$Da_{\mathrm{ads}}=\frac{\tau_{\mathrm{rxn}}}{\tau_{\mathrm{ads}}}=\frac{k_{d}\left(\kappa c_{\mathrm{PA}}+1\right)\left(1+R_{o}/3\delta \right)}{2k_{f1}\Gamma_{\mathrm{PA}}}$$

23 $$Da_{\mathrm{evp}}=\frac{\tau_{\mathrm{rxn}}}{\tau_{\mathrm{evp}}}=\frac{E_{\mathrm{PA}}\left(1+R_{o}/3\delta \right)}{2k_{f1}\Gamma_{\mathrm{PA}}R_{o}^{2}x_{\mathrm{PA}}}$$

Equations 21–23 indicate that droplet radius significantly influences the relative importance of transport processes on reaction kinetics, as later illustrated in Section 3.2. With ([Disp-formula eq17])–([Disp-formula eq19]), the estimated time scale of each process in a microdroplet with an initial radius of 300 μm at two initial PA concentrations *m*_PA,o_ is shown in Table 3. These timescales are highly dependent on *R*_o_, and estimated values over a broader range of *R*_o_ are provided in SI Table S1. The timescales of diffusion and adsorption are significantly shorter than those of reaction and evaporation (Da_dif_ and Da_ads_ are much greater than 1), suggesting that the transport processes of PA and ZA do not serve as limiting factors in the reaction of PA in microdroplets. Rapid adsorption compared to other processes may explain why its inclusion in the model has a minimal impact on reaction kinetics. Furthermore, as later shown in Figure 4, when the initial PA concentration *m*_PA,o_ = 4.5 mol kg^–1^, the surface and bulk PA concentrations are similar. This further justifies why incorporating adsorption into the model yields reaction rate predictions comparable to those of our previous model, which treats surface and bulk concentrations as the same.^18^

**Table 3:** Timescales of Individual Processes (min)

*m*_PA,o_	τ_dif_	τ_ads_	τ_evp_	τ_rxn_
4.5 mol kg^–1^	8.3	6 × 10^–6^	130	700
2 mol kg^–1^	8.3	1 × 10^–5^	300	1700

**Figure 4:**
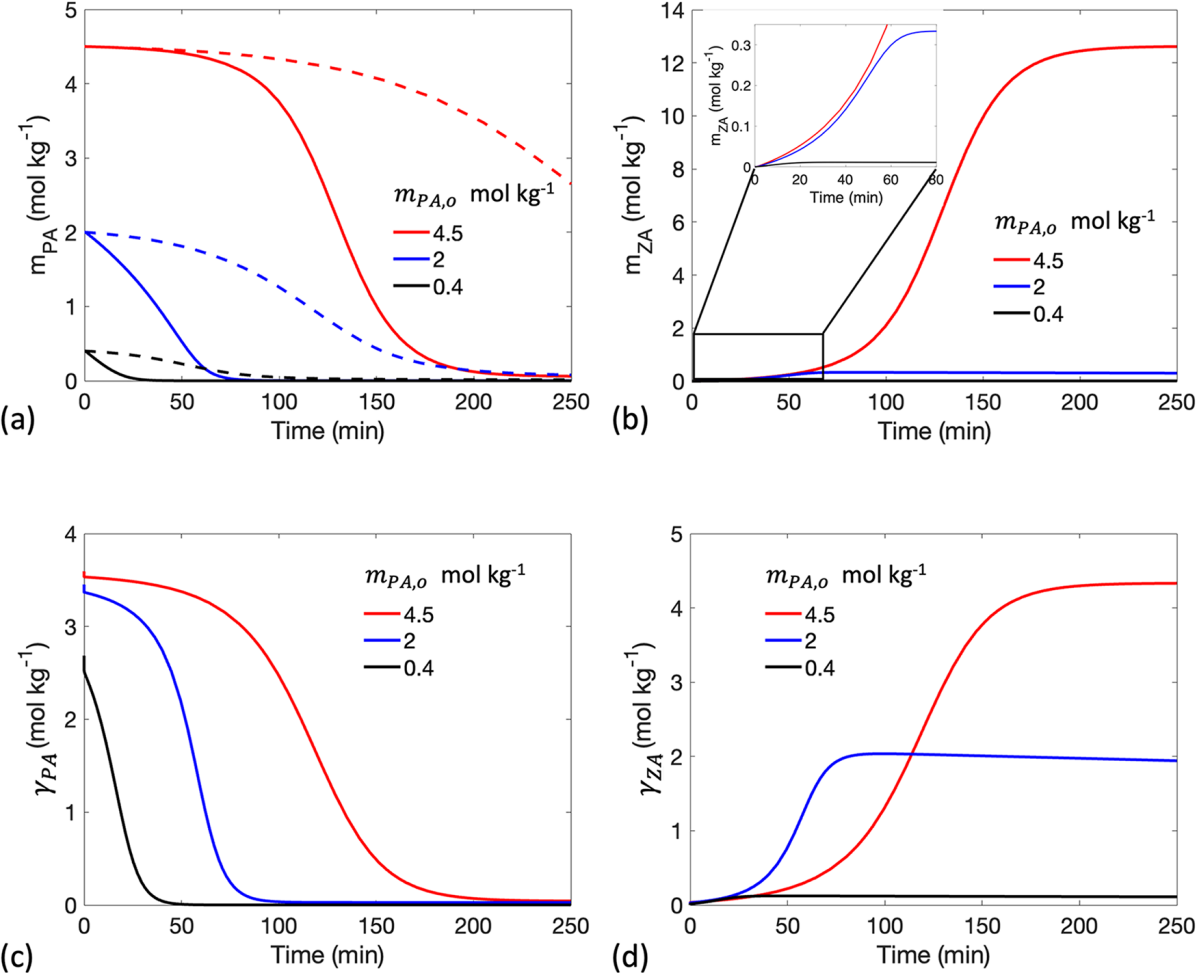
Time evolution of (a) PA bulk concentration *m*_PA_, (b) ZA bulk concentration *m*_ZA_, (c) PA interfacial concentration γ_PA_, and (d) ZA interfacial concentration γ_ZA_ at different initial PA bulk concentrations *m*_PA,o_. The dashed lines in (a) represent the case in which PA evaporation is turned off. Here *R*_o_ = 300 μm. Curves increase in *m*_PA,o_ from the bottom to the top.

#### 3.1.1 Elimination of Induction by Reducing Initial PA Concentration

The model can provide information about the bulk *m*_*X*_ and surface concentrations γ_*X*_ of PA and ZA at different initial PA bulk concentrations *m*_PA,o_. Figure 4a demonstrates that a decrease in *m*_PA,o_ leads to a reduction in the induction period and diminishes the sigmoidal shape of *m*_PA_. However, when PA evaporation is turned off (represented by the dashed lines), a sigmoidal curve remains evident at lower *m*_PA,o_ suggesting that the attenuation of the sigmoidal shape is primarily due to faster PA evaporation. While the sigmoidal kinetics in *m*_PA_ can be eliminated by faster PA evaporation, our previous work demonstrated that gas-phase partitioning alone can give rise to sigmoidal kinetics, even in the absence of autocatalysis.^18^ Therefore, in systems with evaporation or partitioning, sigmoidal kinetics alone cannot confirm or rule out autocatalysis, although their presence in the absence of partitioning can be indicative. In our previous work we noted that the overall fit of the time-dependent *m*_PA_ concentration profile was better when an autocatalytic step was included.

The product concentration *m*_ZA_ shown in Figure 4b is significantly reduced at lower *m*_PA,o_. The percentage of PA transformed to ZA can be calculated as twice the total amount of ZA at the end of the reaction divided by the initial total amount of PA in the droplet

24 $$\mathrm{PA}\;\mathrm{conversion}=\frac{2\int_{0}^{R}C_{\mathrm{ZA}}2\pi r^{2}dr}{C_{\mathrm{PA},o}2\pi R_{o}^{3}/3}\times 100\%$$

A reduction in *m*_PA,o_ correlates with a decrease in the conversion of PA to ZA. The PA conversion rate is 22% when *m*_PA,o_ = 4.5 mol kg^–1^, 19% when *m*_PA,o_ = 2 mol kg^–1^, and 5% when *m*_PA,o_ = 0.4 mol kg^–1^. The competition between evaporation and reaction is quantified by the ratio of their timescales, Da_evp_ (introduced in ([Disp-formula eq23])), which increases with lower Γ_PA_ and *R*_o_. Therefore, at a lower PA concentration or droplet radius, evaporation becomes faster compared to reaction, leading to more PA consumed through evaporation, compared to the reaction, resulting in a lower conversion rate of PA to ZA.

The interfacial concentrations γ_*X*_ shown in Figure 4c,4d govern both reaction and evaporation rates and impact *m*_*X*_ via adsorption and diffusion. As PA is consumed at the interface by reaction and evaporation, PA in the bulk diffuses toward the near-interface region to adsorb and restore γ_PA_. As ZA is generated at the interface, it desorbs to the near-interface region and diffuses into the bulk. Adsorption and diffusion are not the rate-limiting steps in the microdroplet reactions (Table 3). Consequently, the behavior of *m*_*X*_ closely follows γ_*X*_ (Figure 4), with a minimal radial concentration gradient observed in *m*_*X*_ (SI Figure S1). Note that in the calculation, the initial γ_PA_ is set to the equilibrium interfacial concentration corresponding to *m*_PA,o_. There is a rapid minor decline in γ_PA_ within the first second in Figure 4c, as adsorption quickly establishes a new instantaneous equilibrium between the bulk and interface in the presence of reaction, diffusion, and evaporation processes.

#### 3.1.2 Key Role of Adsorption/Desorption in Reaction Kinetics

In the following calculations, we use a lower initial concentration well below the CMC, *m*_PA,o_ = 2 mol kg^–1^. To investigate how the adsorption process influences reaction kinetics, here we systematically varied the ratio of the adsorption rate constant to the desorption rate constant κ_*X*_, and desorption rate constant *k*_d,*X*_ across a range of common surfactants. Note that *k*_d,*X*_ of different organics remains relatively constant, but κ_*X*_ can vary by four to 5 orders of magnitude.^39^

We begin by holding *k*_d,*X*_ constant while varying κ_*X*_ (Figure 5). A higher κ_*X*_ indicates that adsorption is faster than desorption, leading to a greater affinity for the interface and enhanced surfactant properties. A higher κ_*X*_ leads to a higher γ_PA_ at early times (Figure 5c), which accelerates both reaction and evaporation and results in a higher final *m*_ZA_ (Figure 5b), consistent with ([Disp-formula eq23]). However, the concentration temporal profiles show minimal variation as 10 κ_*X*_ increases further to 10^3^ κ_*X*_ (Figure 5), where nearly all interface sites are occupied (Γ_PA_ + Γ_ZA_)/Γ_∞_ ≈ 1 throughout the process (Figure 6a). Consequently, further increases in κ_*X*_ do not significantly increase γ_PA_. Therefore, the key role of κ_*X*_ in surface reaction kinetics is to determine the amount of PA at the surface available to react.

**Figure 5:**
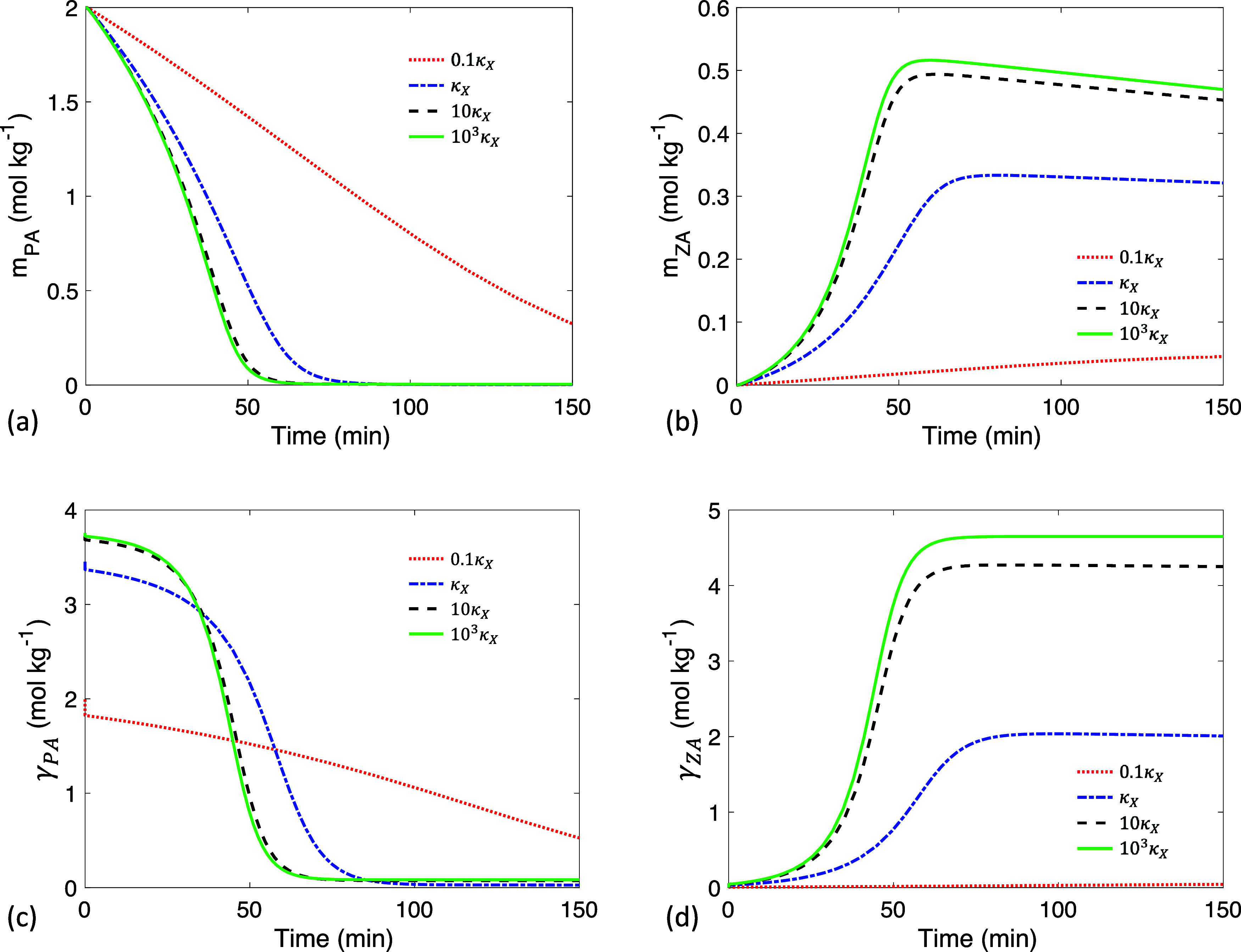
Time evolution of (a) PA bulk concentration *m*_PA_, (b) ZA bulk concentration *m*_ZA_, (c) PA interfacial concentration γ_PA_, and (d) ZA interfacial concentration γ_ZA_ at different ratios of the adsorption rate to the desorption rate κ_*X*_. Here *R*_o_ = 300 μm, *k*_d,*X*_ = 90 s^–1^, and the values of κ_*X*_ are given in Table 2.

**Figure 6:**
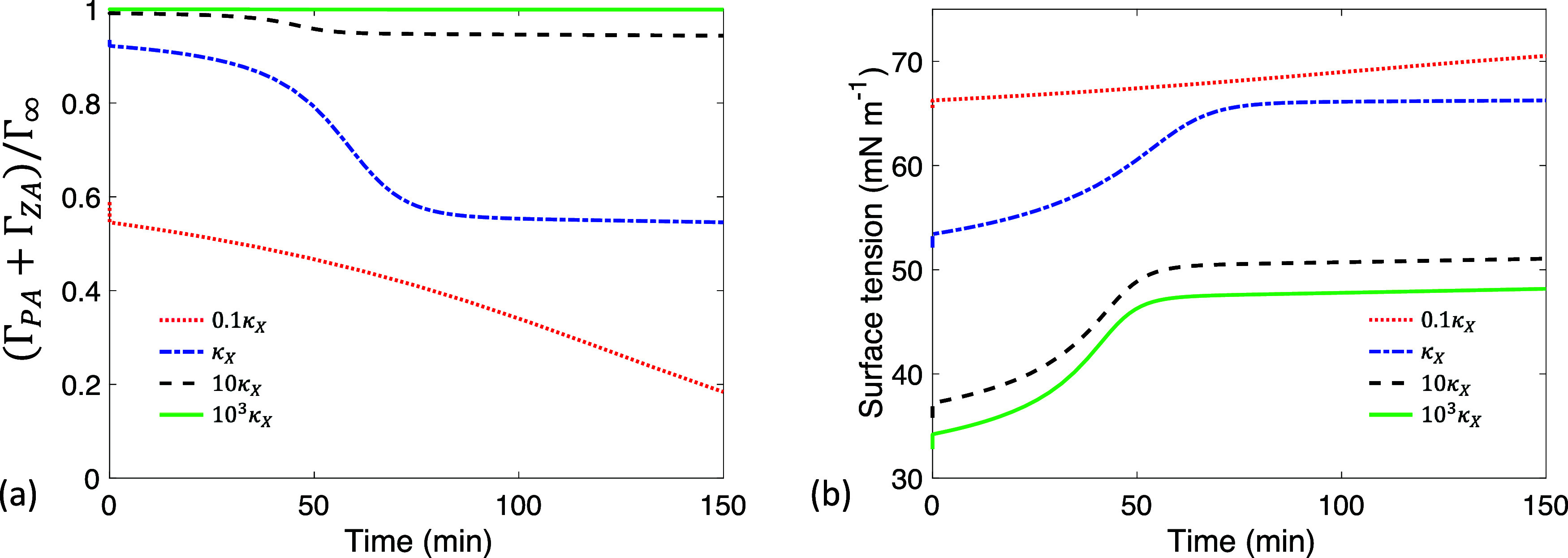
Time evolution of (a) surface coverage (Γ_PA_ + Γ_ZA_)/Γ_∞_ and (b) surface tension at different ratios of the adsorption rate to desorption rate κ_*X*_. Here *R*_o_ = 300 μm and *k*_d,*X*_ = 90 s^–1^.

Interfacial concentrations provide insights into the surface tension σ of a reacting droplet via the Langmuir isotherm

25 $$\sigma =\sigma_{o}+\mathrm{RT}\Gamma_{\infty }\;\ln\left(1-\frac{\Gamma_{\mathrm{PA}}+\Gamma_{\mathrm{ZA}}}{\Gamma_{\infty }}\right)$$

Surface tension increases during the reaction (Figure 6b), due to a reduction in surface coverage (Figure 6a) and the transformation of the surface-active PA into the less surface-active ZA (Figure 2).

Next, parameter κ_*X*_ is kept constant while *k*_d,*X*_ is varied, resulting in slower adsorption (*k*_a,*X*_ = κ_*X*_ *k*_d,*X*_) and desorption as *k*_d,*X*_ decreases. At 0.1 *k*_d,*X*_ there is a sharp decrease in γ_PA_ (Figure 7c) due to slow adsorption of PA, and a sharp increase in γ_ZA_ (Figure 7d) due to slow desorption of ZA. The depletion of PA at the interface at 0.1 *k*_d,*X*_ slows both reaction and evaporation, leading to a slower decrease in *m*_PA_ (Figure 7a) and a higher final value of *m*_ZA_ (Figure 7b). The slow adsorption behavior observed at 0.1 *k*_d,*X*_ (indicating both adsorption and desorption are ten times slower) and 0.1 κ_*X*_ (where only adsorption is ten times slower) can result in distinct kinetics compared to fast adsorption, as demonstrated in Figures 5 and 7.

**Figure 7:**
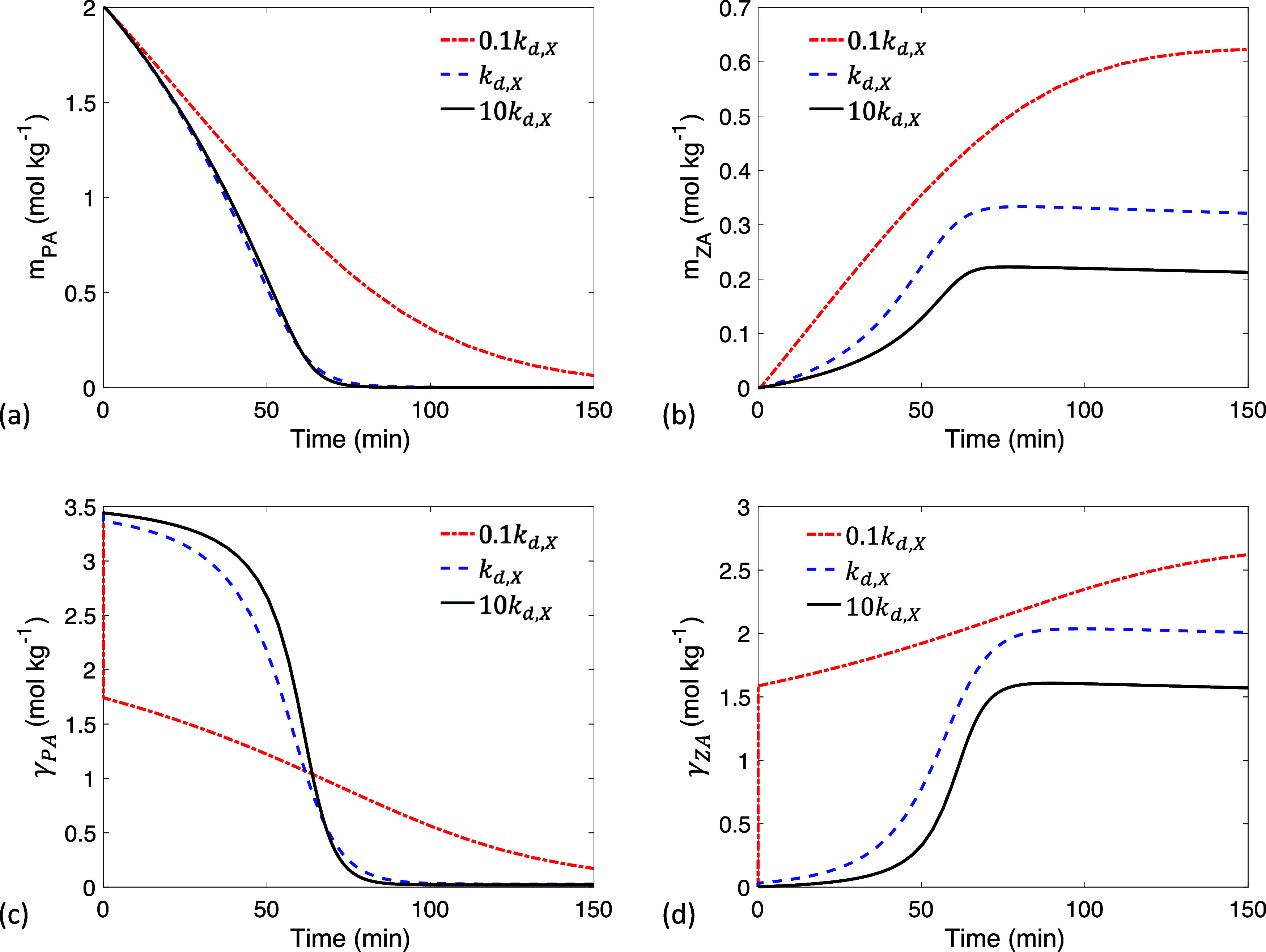
Time evolution of (a) PA bulk concentration *m*_PA_, (b) ZA bulk concentration *m*_ZA_, (c) PA interfacial concentration γ_PA_, and (d) ZA interfacial concentration γ_ZA_ at different desorption rate constants *k*_d,*X*_ with an initial droplet radius *R*_o_ = 300 μm.

### 3.2 Kinetics of Chemical Reactions in Nanometer and Millimeter-Scale Droplets

In Section 3.1, we focused on the reaction kinetics of microscale droplets. Reactions in aerosols can occur at the nanoscale, while those in emulsions involve droplets on the millimeter scale, highlighting the significance of considering a broader range of droplet sizes in reaction studies. Equations 18 and 19 suggest that the relative significance of physical and chemical processes is strongly influenced by the size of the droplets. We set the initial PA concentration *m*_PA,o_ = 2 mol kg^–1^ and solve for the critical radius where the timescales of the two processes become comparable. There are four critical radii as illustrated in Figure 8: two at the nanometer scale, associated with adsorption, diffusion, and evaporation, and two at the millimeter scale, related to reaction, evaporation, and diffusion. For example, *R*_ads,evp_ is the critical radius where the timescales of adsorption and evaporation are comparable. Progressing from the top to the bottom of the table, the timescales of the processes increase, indicating that the slower processes increasingly act as limiting factors. The estimated timescales for droplets with radii selected from each of the five regions, divided by the four critical radii, are listed in SI Table S1. The relative magnitudes of these timescales clearly follow the trends shown in Figure 8.

**Figure 8:**
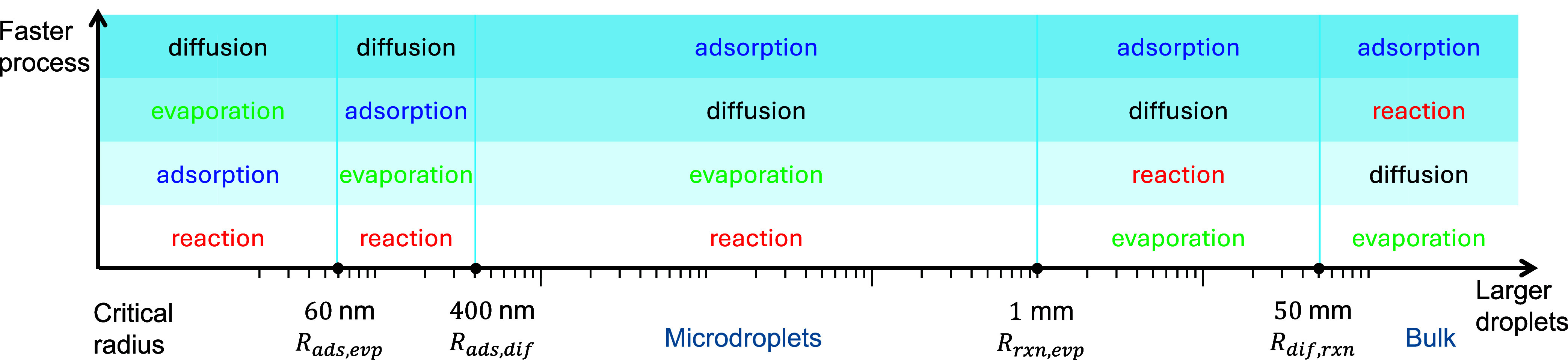
Different processes dominate the kinetics of droplet chemistry on different scales. Note that the rate-limiting steps are the slowest processes; the processes in the bottom rows are the more important ones for the given size scale.

Note that here the diffusion of PA in the droplet is always much faster than PA evaporation, consistent with the assumption in the PA gas-phase diffusion-limited evaporation model described by ([Disp-formula eq12]). Additionally, adsorption is always faster than the reaction for PA. Figure 8 shows that adsorption and diffusion are not the rate-limiting steps of the PA reaction in microdroplets. However, diffusion plays a significant role in millimeter-scale droplets (Section 3.2.1), while adsorption becomes more critical in nanodroplets (Section 3.2.2).

#### 3.2.1 Diffusion-Limited in Millimeter-Scale Droplets

The rate-limiting effect of diffusion becomes increasingly significant in millimeter-scale droplets, particularly when the droplet radius exceeds the critical threshold of *R*_dif,rxn_ = 50 mm (Figure 8). The kinetics of droplets with *R*_o_ = 2 mm and *R*_o_ = 10 mm are shown in Figure 9. The solid lines represent the results from our model detailed in Section 2, while the dashed lines represent results obtained using a “well-mixed” model (detailed in SI). In the well-mixed model, diffusion is assumed to be rapid enough to eliminate radial concentration gradients, resulting in a uniform bulk concentration throughout the droplet. In Figure 9, minimal diffusion limitation is observed in the case *R*_o_ = 2 mm, where the solid and dashed lines align closely, but not for droplets with *R*_o_ = 10 mm. In the diffusion-limited case (*R*_o_ = 10 mm), the greater diffusion distance in larger droplets results in a longer time for PA to travel from the deeper bulk to the subsurface, where it can adsorb to the surface. Slow replenishment of PA near the surface delays adsorption, leading to a faster decline in γ_PA_ compared to *m*_PA_ (Figure 9a,c) and a depletion of *m*_PA_ near the interface in larger droplets (Figure 10a). In contrast, Γ_ZA_ increases faster than *m*_ZA_ (Figure 9b,d). The slow diffusion of ZA from the subsurface to the deeper bulk results in a slower increase in *m*_ZA_ and the accumulation of ZA near the interface (Figure 10b). Previous literature also reported concentration gradients of reactants and products along the radius as a result of slow diffusion, identifying diffusion as a rate-limiting factor in the reaction.^34,36^

**Figure 9:**
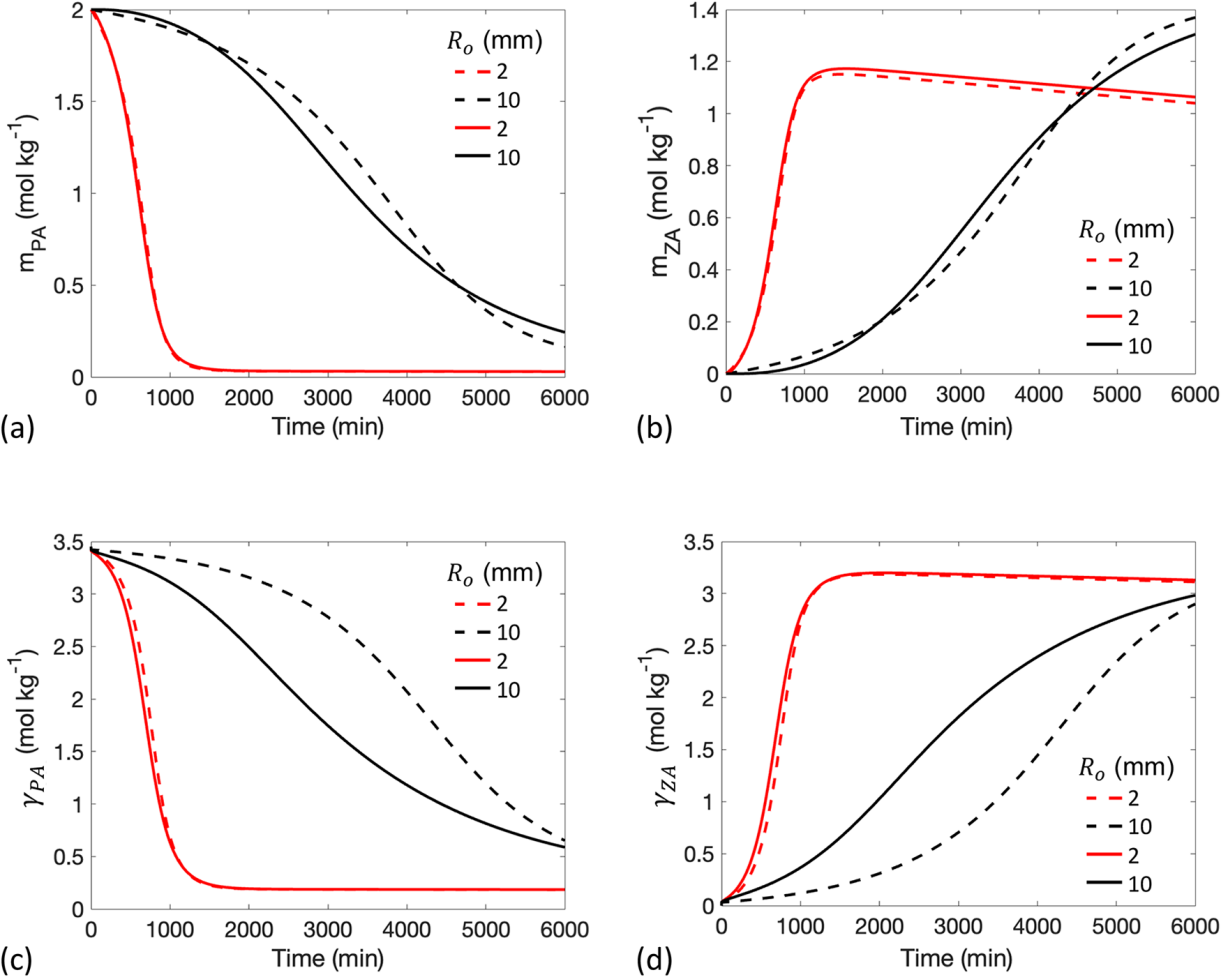
Time evolution of (a) PA bulk concentration *m*_PA_, (b) ZA bulk concentration *m*_ZA_, (c) PA interfacial concentration γ_PA_, and (d) ZA interfacial concentration γ_ZA_ with initial droplet radii *R*_o_ = 2 mm and *R*_o_ = 10 mm. The dashed lines represent the well-mixed model, where the diffusion time is assumed to be fast enough to be negligible. Curves increase in *R*_o_ from left to right.

**Figure 10:**
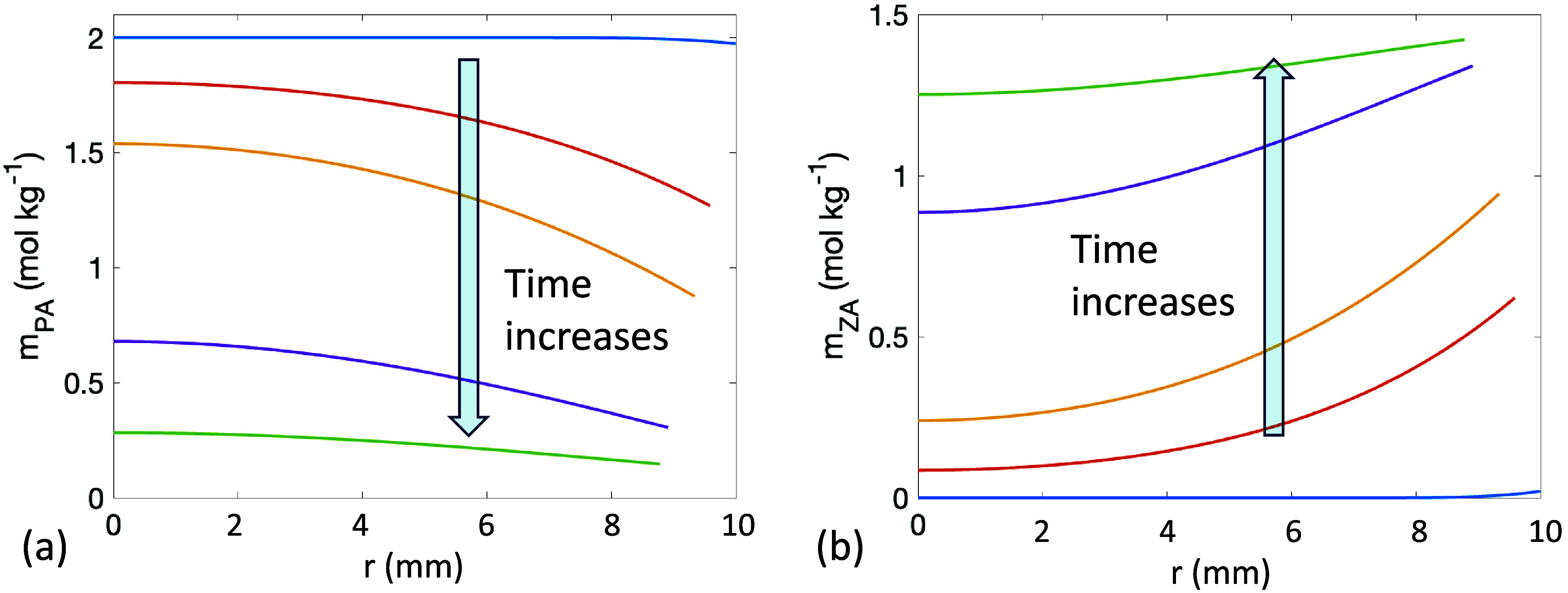
(a) *m*_PA_ and (b) *m*_ZA_ along the radius *r* at 5 different times with *R*_o_ = 10 mm.

#### 3.2.2 Adsorption-Limited in Nanometer-Scale Droplets

Adsorption can be a rate-limiting process in nanodroplets (Figure 8). The time evolution of various quantities for droplets with *R*_o_ = 50 nm and *R*_o_ = 200 nm is shown in Figure 11. When adsorption is rate-limiting, there is a rapid decrease in γ_PA_ (Figure 11c) and a sharp increase in γ_ZA_ at the beginning (Figure 11d), behavior also observed in the slow adsorption case (0.1 *k*_d_) shown in Figure 7. Adsorption is insufficient to replenish consumed PA, while desorption is insufficient to release produced ZA. Moreover, as shown in Figure 8 and indicated by the low Da_evp_ values at small *R* in eq 23, PA evaporates significantly faster than it reacts in nanodroplets. As a result, the majority of PA is lost to evaporation rather than to reaction, leading to a substantially reduced final *m*_ZA_ (Figure 11b) and minimal reaction activity. Rapid evaporation also eliminates the induction period in nanodroplets (Figure 11a), and the PA evolution follows a curve similar to the experiment by Kim et al.^34^ where *R*_o_ = 240 nm.

**Figure 11:**
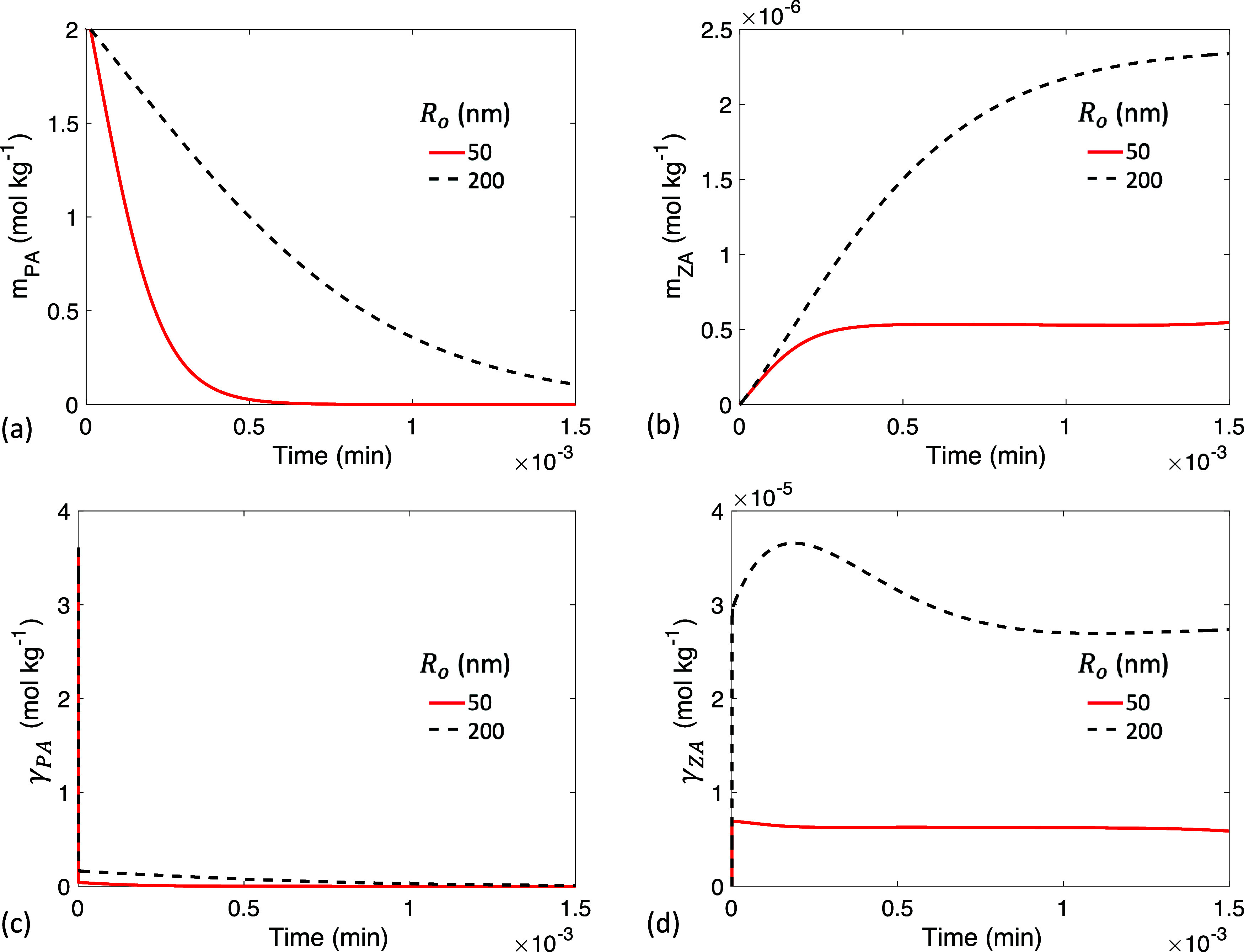
Time evolution of (a) PA bulk concentration *m*_PA_, (b) ZA bulk concentration *m*_ZA_, (c) PA interfacial concentration γ_PA_, and (d) ZA interfacial concentration γ_ZA_ with initial droplet radii *R*_o_ = 50 nm and *R*_o_ = 100 nm.

A tradeoff between the reaction rate and PA conversion efficiency is revealed by Figures 9 and 11. In millimeter-scale droplets (Figure 9), reactions proceed over several hours due to a low surface-to-volume ratio and slow diffusion, which limit the reaction rate. Nevertheless, evaporation is even slower in these millimeter droplets, allowing more PA to convert to ZA (resulting in a high *m*_ZA_ value in Figure 9b) rather than being lost to evaporation. In contrast, in nanometer-scale droplets (Figure 11), the reaction completes in less than one s, but nearly all PA evaporates before reactions occur, leaving *m*_ZA_ close to zero. SI Figure S2 shows the conversion rate as a function of reaction time and droplet radius, where the reaction time is defined as the time required for the PA concentration to decrease to 90% of its initial value. The conversion rate increases with droplet size, but the improvement becomes less significant beyond 200 μm, with the conversion remaining relatively high. Similarly, the increase in reaction time with droplet radius is modest, below 2000 μm. These trends suggest a potential optimal droplet size between 200 and 2000 μm. For microdroplet reactor design, this range offers a balance between fast reaction kinetics and high conversion efficiency, indicating that the optimal droplet size lies within the micrometer scale.

## 4 Conclusions

This study presents a model that characterizes the interplay among diffusion, adsorption, evaporation, and reaction in a microdroplet, highlighting the competition and impact on reaction kinetics. Using the condensation reaction of pyruvic acid to zymonic acid as an example, our model successfully predicts experimental observations of the concentration dynamics and droplet size evolution. We also showed how the kinetic behavior of this (or any) reaction in microdroplets would change for different values of various kinetic and thermodynamic parameters.

While our model is based on experiments with microdroplets in a controlled humidity cell, where water partitioning is controlled, the water partitioning is generally rapid compared with other processes. Therefore, the general principles governing the interplay between interfacial transport processes and reaction are relevant to other droplet generation methods, such as electrospray or nebulization. However, additional processes such as advection or charge may also play a role and could be important aspects in future work. The current framework can also be extended to more complex systems with multiple reactants by incorporating additional surface and bulk species, competitive adsorption via modified isotherms, and salting-out effects through ionic-concentration-dependent adsorption or partitioning models. These additions are compatible with the model and broaden its applicability to multicomponent or high-salinity environments.

Overall, our findings indicate that while diffusion and adsorption are not rate-limiting in microdroplets, diffusion affects kinetics at the millimeter scale and adsorption plays a role at the nanometer scale. Additionally, competition between reaction and evaporation highlights the critical role of droplet size in the tradeoff between reaction rates and PA conversion percentage. These results underscore the complexity of microdroplet reaction kinetics, emphasizing the central role of interfacial processes. The insights provided here establish a general framework for understanding competing processes in reacting microdroplets and “the rules of microdroplet reactivity” in that our framework offers practical guidelines for optimizing droplet size to enhance reaction efficiency.

## Supplementary Material



## Notes

More details and equations for the well-mixed model; timescales of individual processes (min) for *m*_PA,o_ = 2 mol kg^–1^ (Table S1); kinetic modeling of the autocatalytic condensation reaction of PA in aqueous microdroplets (Figure S1); and dependence of conversion rate on initial droplet radius *R*_o_ and reaction time (Figure S2) (PDF)

## References

[1] Rovelli G., Jacobs M. I., Willis M. D., Rapf R. J., Prophet A. M., Wilson K. R. (2020). A critical analysis of electrospray techniques for
the determination of accelerated rates and mechanisms of chemical
reactions in droplets. Chem. Sci..

[2] Angle K. J., Neal E. E., Grassian V. H. (2021). Enhanced
rates of transition-metal-ion-catalyzed
oxidation of S­(IV) in aqueous aerosols: Insights into sulfate aerosol
formation in the atmosphere. Environ. Sci. Technol..

[3] Wei Z., Li Y., Cooks R. G., Yan X. (2020). Accelerated reaction kinetics in
microdroplets: Overview and recent developments. Annu. Rev. Phys. Chem..

[4] Wilson K. R., Prophet A. M., Rovelli G., Willis M. D., Rapf R. J., Jacobs M. I. (2020). A kinetic description
of how interfaces accelerate
reactions in micro-compartments. Chem. Sci..

[5] Yan X., Bain R. M., Cooks R. G. (2016). Organic
reactions in microdroplets:
reaction acceleration revealed by mass spectrometry. Angew. Chem., Int. Ed..

[6] Li M., Boothby C., Continetti R. E., Grassian V. H. (2023). Size-Dependent Sigmoidal
Reaction Kinetics for Pyruvic Acid Condensation at the Air-Water Interface
in Aqueous Microdroplets. J. Am. Chem. Soc..

[7] Edwards M. Q., Holden D. T., Cooks R. G. (2025). Abiotic
formation of hexoses and
disaccharides in aqueous microdroplets. Chem.
Sci..

[8] Nandy A., Kumar A., Mondal S., Koner D., Banerjee S. (2023). Spontaneous
generation of aryl carbocations from phenols in aqueous microdroplets:
aromatic SN1 reactions at the air-water interface. J. Am. Chem. Soc..

[9] Kumar A., Avadhani V. S., Nandy A., Mondal S., Pathak B., Pavuluri V. K. N., Avulapati M. M., Banerjee S. (2024). Water microdroplets
in air: a hitherto unnoticed natural source of nitrogen oxides. Anal. Chem..

[10] Gong K., Nandy A., Song Z., Li Q.-S., Hassanali A., Cassone G., Banerjee S., Xie J. (2024). Revisiting
the Enhanced
Chemical Reactivity in Water Microdroplets: The Case of a Diels–Alder
Reaction. J. Am. Chem. Soc..

[11] Nandy A., Mondal S., Koner D., Banerjee S. (2024). Heavy water microdroplet
surface enriches the lighter isotopologue impurities. J. Am. Chem. Soc..

[12] Nandy A., Murmu G., Rana A., Saha S., Banerjee S. (2025). Sprayed Microdroplets
Architect a Polyoxometalate Framework. Angew.
Chem..

[13] Meng Y., Xia Y., Xu J., Zare R. N. (2025). Spraying of water microdroplets forms
luminescence and causes chemical reactions in surrounding gas. Sci. Adv..

[14] Song X., Basheer C., Xia Y., Li J., Abdulazeez I., Al-Saadi A. A., Mofidfar M., Suliman M. A., Zare R. N. (2023). One-step
formation of urea from carbon dioxide and nitrogen using water microdroplets. J. Am. Chem. Soc..

[15] Meng Y., Zare R. N., Gnanamani E. (2024). Superfast Formation of C (sp2)- N,
C (sp2)- P, and C (sp2)- S Vinylic Bonds in Water Microdroplets. Angew. Chem., Int. Ed..

[16] Graedel T. E., Weschler C. (1981). Chemistry within aqueous atmospheric aerosols and raindrops. Rev. Geophys..

[17] Prather K. A., Hatch C. D., Grassian V. H. (2008). Analysis of atmospheric aerosols. Annu. Rev. Anal. Chem..

[18] Li M., Yang S., Rathi M., Kumar S., Dutcher C. S., Grassian V. H. (2024). Enhanced condensation
kinetics in aqueous microdroplets
driven by coupled surface reactions and gas-phase partitioning. Chem. Sci..

[19] Houle F. A., Wiegel A. A., Wilson K. R. (2018). Changes in reactivity as chemistry
becomes confined to an interface. The case of free radical oxidation
of C30H62 alkane by OH. The. J. Phys. Chem.
Lett..

[20] Agresti J. J., Antipov E., Abate A. R., Ahn K., Rowat A. C., Baret J.-C., Marquez M., Klibanov A. M., Griffiths A. D., Weitz D. A. (2010). Ultrahigh-throughput screening in drop-based microfluidics
for directed evolution. Proc. Natl. Acad. Sci.
U.S.A..

[21] Urban P. L., Goodall D. M., Bruce N. C. (2006). Enzymatic microreactors in chemical
analysis and kinetic studies. Biotechnol. Adv..

[22] Thickett S. C., Gilbert R. G. (2007). Emulsion polymerization:
State of the art in kinetics
and mechanisms. Polymer.

[23] Lovell P. A., Schork F. J. (2020). Fundamentals of
emulsion polymerization. Biomacromolecules.

[24] Guzman M. I., Martin S. T. (2009). Prebiotic metabolism:
production by mineral photoelectrochemistry
of *α*-ketocarboxylic acids in the reductive
tricarboxylic acid cycle. Astrobiology.

[25] Cooper G., Reed C., Nguyen D., Carter M., Wang Y. (2011). Detection
and formation scenario of citric acid, pyruvic acid, and other possible
metabolism precursors in carbonaceous meteorites. Proc. Natl. Acad. Sci. U.S.A..

[26] Griffith E. C., Carpenter B. K., Shoemaker R. K., Vaida V. (2013). Photochemistry of aqueous
pyruvic acid. Proc. Natl. Acad. Sci. U.S.A..

[27] Bissette A. J., Fletcher S. P. (2013). Mechanisms of autocatalysis. Angew. Chem., Int. Ed..

[28] Hanopolskyi A. I., Smaliak V. A., Novichkov A. I., Semenov S. N. (2021). Autocatalysis: kinetics,
mechanisms and design. ChemSystemsChem.

[29] Jacobs M. I., Johnston M. N., Mahmud S. (2024). Exploring How the Surface-Area-to-Volume
Ratio Influences the Partitioning of Surfactants to the Air-Water
Interface in Levitated Microdroplets. J. Phys.
Chem. A.

[30] Xiong H., Lee J. K., Zare R. N., Min W. (2020). Strong concentration
enhancement of molecules at the interface of aqueous microdroplets. J. Phys. Chem. B.

[31] Ruiz-López M. F., Martins-Costa M. T. (2022). Disentangling reaction rate acceleration
in microdroplets. Phys. Chem. Chem. Phys..

[32] LaCour R. A., Heindel J. P., Zhao R., Head-Gordon T. (2025). The Role of
Interfaces and Charge for Chemical Reactivity in Microdroplets. J. Am. Chem. Soc..

[33] Chen C. J., Williams E. R. (2023). The role of analyte concentration in accelerated reaction
rates in evaporating droplets. Chem. Sci..

[34] Kim P., Reynolds R. S., Deal A. M., Vaida V., Ahmed M., Wilson K. R. (2024). Accelerated zymonic
acid formation from pyruvic acid
at the interface of aqueous nanodroplets. J.
Phys. Chem. Lett..

[35] Wilson K. R., Prophet A. M. (2024). Chemical kinetics in microdroplets. Annu. Rev. Phys. Chem..

[36] Fallah-Araghi A., Meguellati K., Baret J.-C., Harrak A. E., Mangeat T., Karplus M., Ladame S., Marques C. M., Griffiths A. D. (2014). Enhanced
chemical synthesis at soft interfaces: A universal reaction-adsorption
mechanism in microcompartments. Phys. Rev. Lett..

[37] Zhong J., Kumar M., Anglada J., Martins-Costa M. T. C., Ruiz-Lòpez M.
F., Zeng X. C., Francisco J. S. (2019). Atmospheric
spectroscopy and photochemistry at environmental water interfaces. Annu. Rev. Phys. Chem..

[38] Duff D. G., Ross S. M., Vaughan D. H. (1988). Adsorption
from solution: An experiment
to illustrate the Langmuir adsorption isotherm. J. Chem. Educ..

[39] Bleys G., Joos P. (1985). Adsorption kinetics
of bolaform surfactants at the air/water interface. J. Phys. Chem. A.

[40] Perkins R. J., Shoemaker R. K., Carpenter B. K., Vaida V. (2016). Chemical equilibria
and kinetics in aqueous solutions of zymonic acid. J. Phys. Chem. A.

[41] Cazabat A.-M., Guena G. (2010). Evaporation of macroscopic sessile
droplets. Soft Matter.

[42] Armstrong S., McHale G., Ledesma-Aguilar R., Wells G. G. (2019). Pinning-free evaporation
of sessile droplets of water from solid surfaces. Langmuir.

[43] Raznjevic, K. Handbook of Thermodynamic Tables and Charts; Hemisphere Publishing Corp.: Washington, DC, 1976.

[44] Frenkel, M. ; Kabo, G. J. ; Marsh, K. N. ; Roganov, G. N. ; Wilhoit, R. C. Thermodynamics of Organic Compounds in the Gas State; CRC Press, 1994; Vol. 1.

[45] Stokes R. H., Robinson R. (1966). Interactions in aqueous nonelectrolyte solutions. I.
Solute-solvent equilibria. J. Phys. Chem. A.

[46] Zuend A., Marcolli C., Booth A., Lienhard D. M., Soonsin V., Krieger U., Topping D. O., McFiggans G., Peter T., Seinfeld J. H. (2011). New and extended
parameterization
of the thermodynamic model AIOMFAC: calculation of activity coefficients
for organic-inorganic mixtures containing carboxyl, hydroxyl, carbonyl,
ether, ester, alkenyl, alkyl, and aromatic functional groups. Atmos. Chem. Phys..

[47] Alvarez N. J., Walker L. M., Anna S. L. (2010). Diffusion-limited
adsorption to a
spherical geometry: The impact of curvature and competitive time scales. Phys. Rev. E.

