# Supplementary material for: Role of Interfacial Processes in Accelerated Reactions in Nano- and Microdroplets
Source: J Phys Chem A. 2025 Jul 4;129(28):6424–36. doi: 10.1021/acs.jpca.5c03287 (PMC12278221; doi:10.1021/acs.jpca.5c03287)
Supplement: Supplementary file 1 [file jp5c03287_si_001.pdf]

Supporting Information (SI) for The Role of Interfacial Processes in Accelerated Reaction in Nano- and Micro-droplets

Shu Yang,[†] Meng Li,[‡] Justin Wang,[‡] Vicki H. Grassian,[‡] Satish Kumar,[*,¶] and Cari S. Dutcher[*,†,¶]

†*Department of Mechanical Engineering, University of Minnesota, Minneapolis, MN 55455, USA*

‡*Department of Chemistry and Biochemistry, University of California San Diego, La Jolla, CA 92093, USA*

¶*Department of Chemical Engineering and Materials Science, University of Minnesota, Minneapolis, MN 55455, USA*

E-mail: kumar030@umn.edu; cdutcher@umn.edu

The SI contains additional details on the manuscript.

The well-mixed model

The well-mixed model assumes a homogeneous droplet concentration in the bulk, based on the assumption that diffusion is significantly faster than other processes, allowing radial concentration gradients to be immediately eliminated. Below, we present the equations for the well-mixed model, noting that only the bulk concentration equations differ from those in the spatially resolved model. Therefore, we provide only the derivation of the bulk concentration equation here. The mass conservation of substance X is expressed as:

$$\frac{d}{dt}\left(C_X\frac{2\pi}{3}R(t)^3\right)+\frac{d}{dt}\left(\Gamma_X 2\pi R(t)^2\delta\right)=2\pi R^2\delta J_{X,rxn}+\frac{dV_{X,evp}}{dt}\frac{1}{\nu_X}. \tag{SI-1}$$

The physical meanings of each term and variable remain the same as in (4). Expanding SI-1 yields the evolution equations for bulk and surface concentrations:

$$\frac{dC_X}{dt}=-\frac{3}{R}C_X\frac{dR}{dt}+J_{X,sorp}, \tag{SI-2}$$

$$\frac{d\Gamma_X}{dt}=-\frac{2}{R}\Gamma_{PA}\frac{dR}{dt}+J_{X,rxn}-J_{X,sorp}+\frac{dV_{X,evp}}{dt}\frac{1}{2\pi R^2\delta\nu_X}. \tag{SI-3}$$

Equations (SI-2) and (SI-3), coupled with size-change equation (14), constitute the well-mixed model.

Table S1: The timescales of individual processes (min) for $m_{PA,o}=2$ mol kg^{-1}.

R_o	τ_{dif}	τ_{ads}	τ_{evp}	τ_{rxn}
10 nm	9.3×10^{-9}	1.2×10^{-5}	3.2×10^{-7}	7.2×10^{-2}
100 nm	9.3×10^{-7}	1.2×10^{-5}	3.2×10^{-5}	5.7×10^{-1}
10 μm	9.3×10^{-3}	1.2×10^{-5}	3.2×10^{-1}	55
500 μm	23	1.2×10^{-5}	8.1×10^{2}	2.8×10^{3}
2 mm	3.7×10^{2}	1.2×10^{-5}	1.3×10^{4}	1.1×10^{4}
100 mm	9.3×10^{5}	1.2×10^{-5}	3.2×10^{7}	5.6×10^{5}

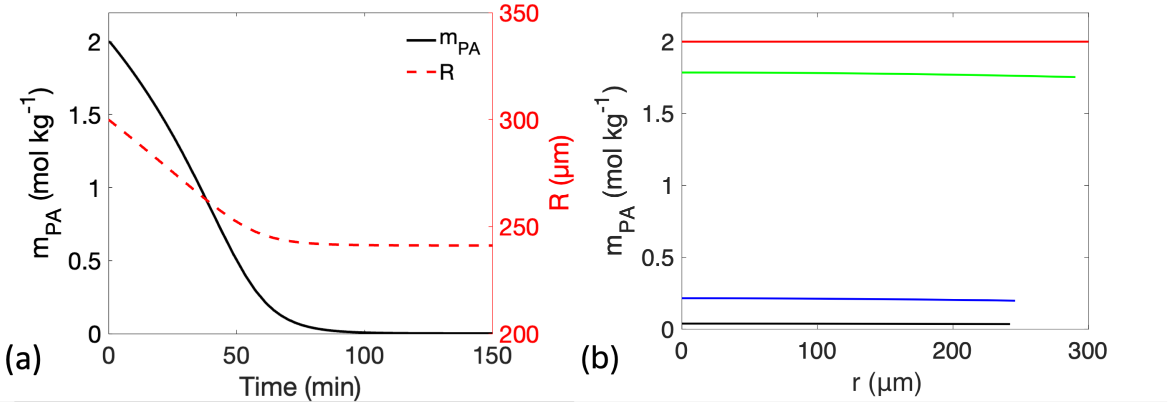

Figure S1: Kinetic modeling of autocatalytic condensation reaction of PA in aqueous microdroplets. (a) Time evolution of m_{PA} at $r = R/2$ and R, and (b) m_{PA} versus radial positions (r) at four different times for a droplet with initial radius R_o=300 μm and initial PA concentration $m_{PA,o} = 2$ mol kg^{-1}.

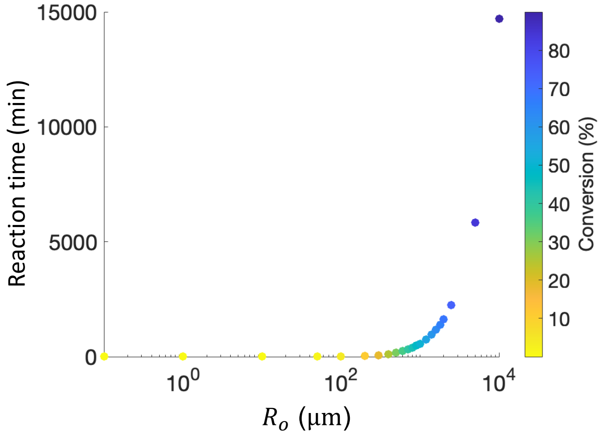

Figure S2: Dependence of conversion rate on initial droplet radius R_o and reaction time. Reaction time is defined as the time required for the PA concentration to decrease to 90% of its initial value. Color coding indicates the conversion rate, calculated using Equation (24).
